# Supplementary material for: Holocentromeres can consist of merely a few megabase-sized satellite arrays
Source: Nat Commun. 2023 Jun 13;14:3502. doi: 10.1038/s41467-023-38922-7 (PMC10264360; doi:10.1038/s41467-023-38922-7)
Supplement: Supplementary file 1 — Supplementary Information [file 41467_2023_38922_MOESM1_ESM.pdf]

Supplementary information

Holocentromeres can consist of merely a few megabase-sized satellite arrays

Kuo et al.,

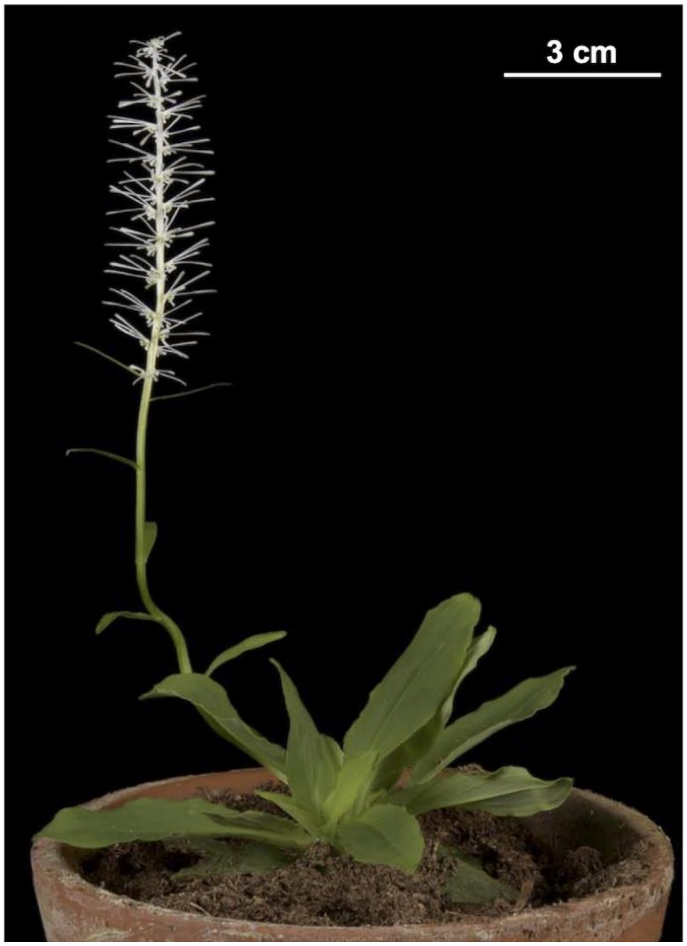

Supplementary Fig. 1. Flowering plant of *C. japonica.*

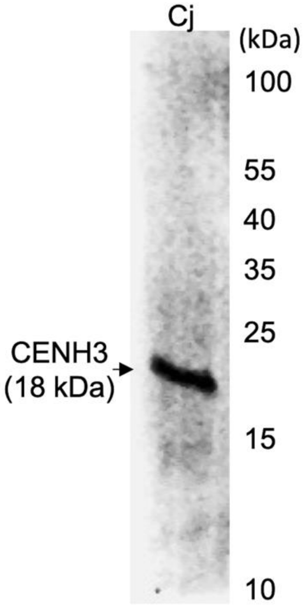

Supplementary Fig. 2.

Western blot analysis of *C. japonica* CENH3. The specificity of the *C. japonica* anti-CENH3 antibody was confirmed by the detection of the predicted 18 kDa nuclear protein. The experiment was performed once. Cj: *C japonica*. Source Data are provided as a Source Data file.

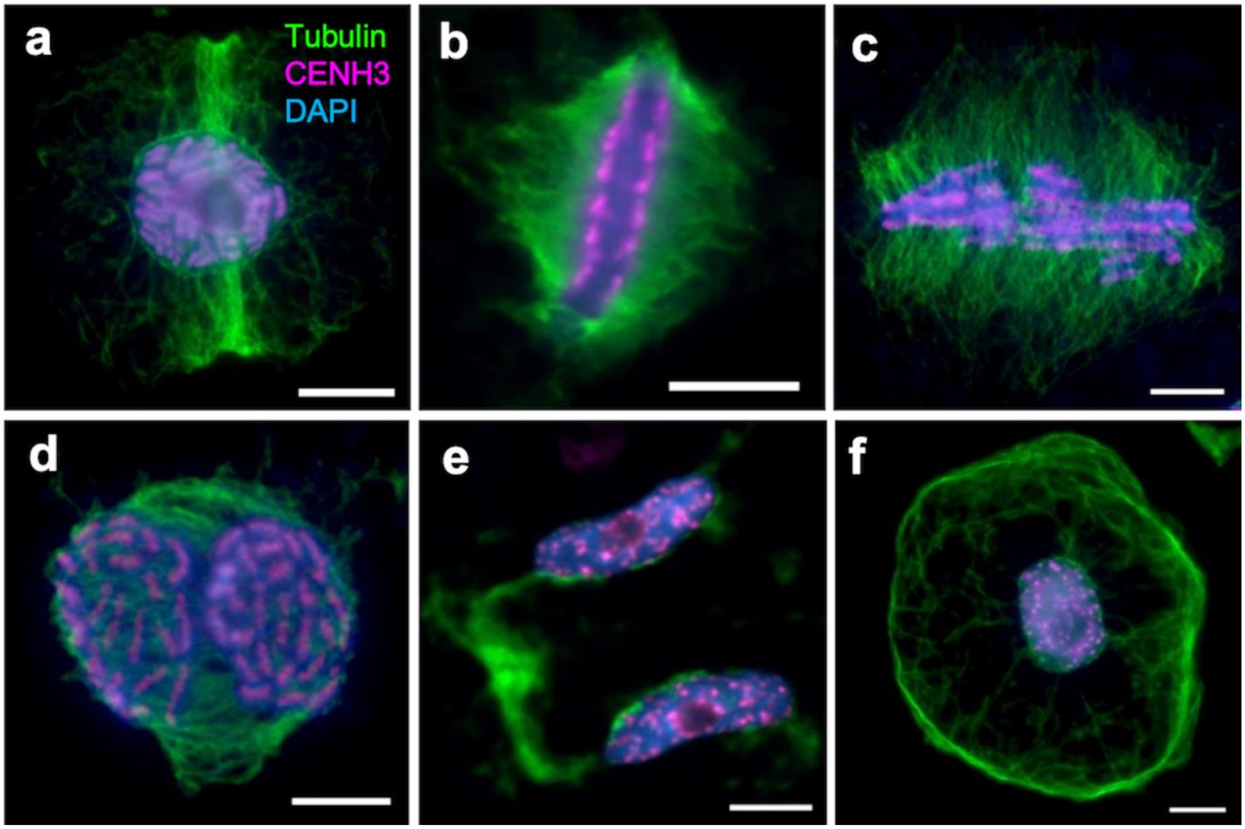

Supplementary Fig. 3.

Immunolabelling of CENH3 (purple) and alpha-tubulin (green) in mitotic (a) prophase, (b) metaphase, (c) anaphase, (d) early telophase, (e) late telophase, and (f) interphase cells of *C. japonica.* Scale bar, 5 µm. (a-f) At least two independent experiments were carried out to confirm the reproducibility of the labeling patterns.

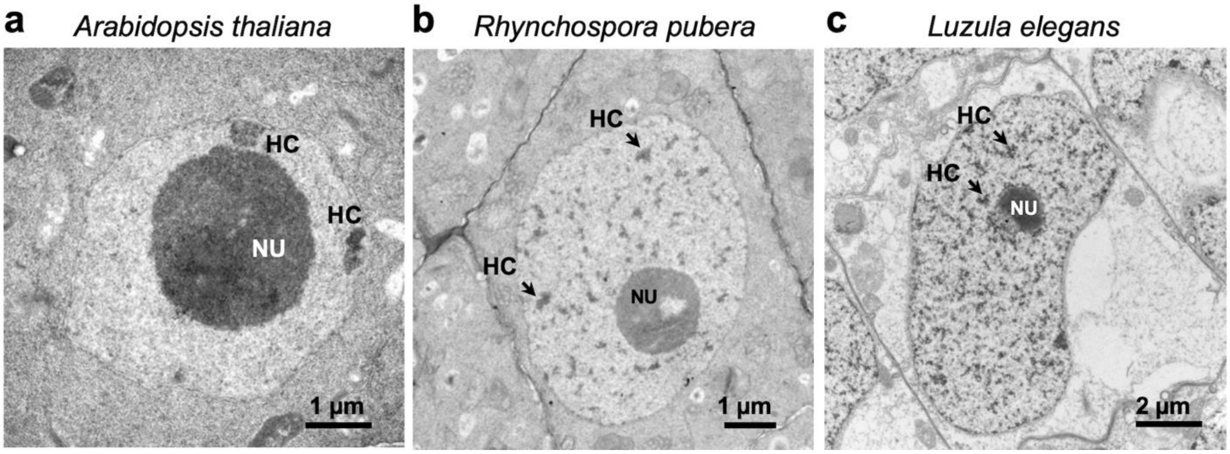

Supplementary Fig. 4.

Transmission electron micrographs of root interphase nuclei of monocentric (a) *A. thaliana* and holocentric (b) *R. pubera* and (c) *L. elegans*. HC: heterochromatic chromocenter (arrows), NU: nucleolus. (a-c) At least two independent experiments were carried out to confirm the reproducibility of the observed structural patterns.

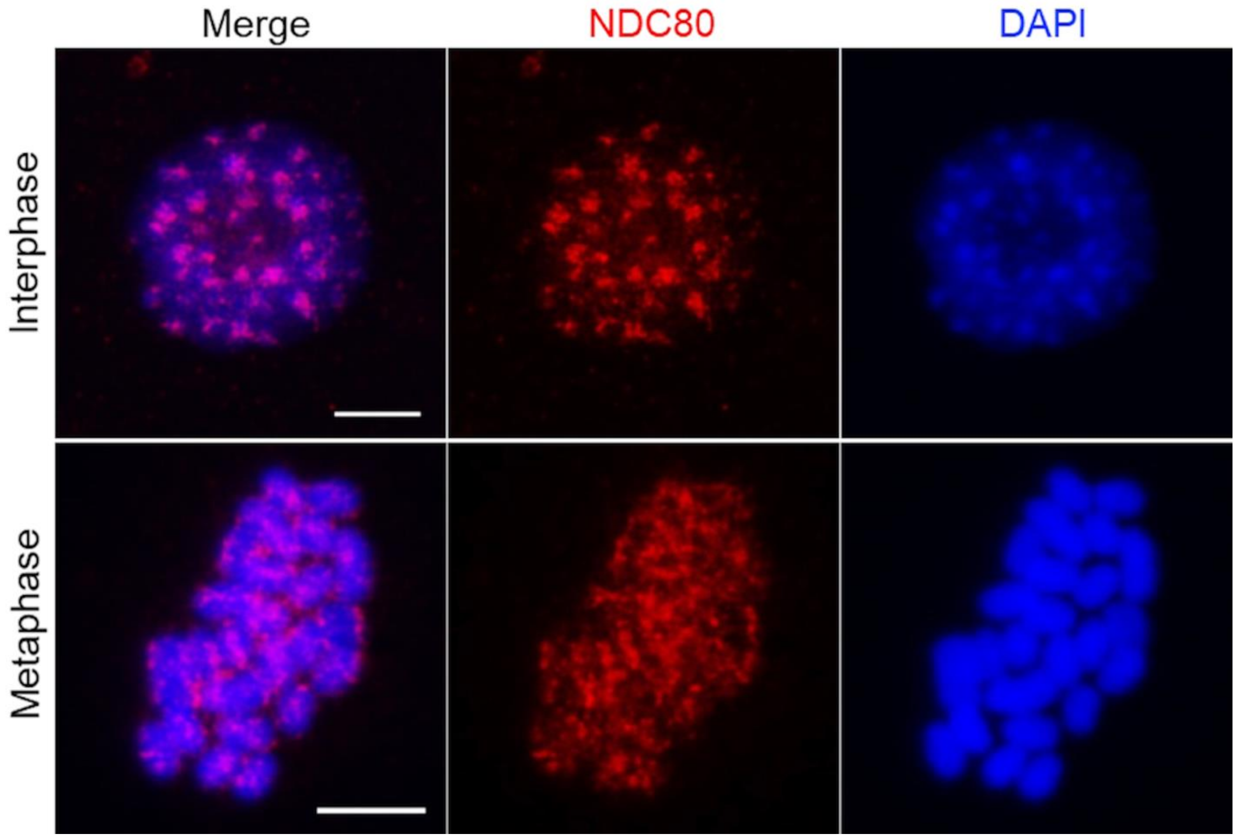

Supplementary Fig. 5.

Immunodetection of the kinetochore protein NDC80 (red) in interphase nucleus and mitotic metaphase chromosomes of *C. japonica*. Nucleus and chromosomes were counterstained with DAPI and pseudocolored in blue. Scale bar, 5 μm. At least two independent experiments were carried out to confirm the reproducibility of the labeling patterns.

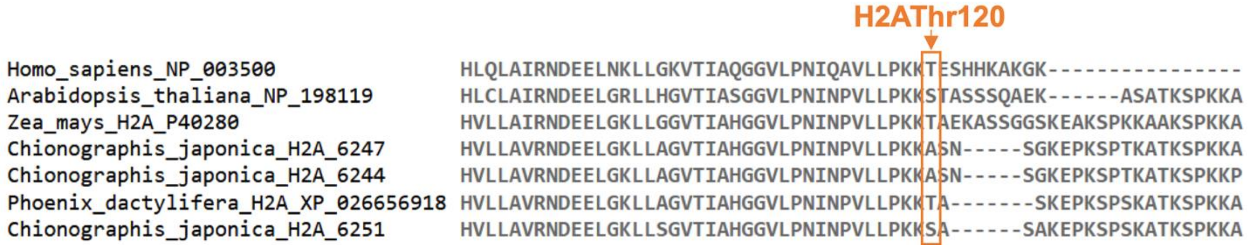

Supplementary Fig. 6.

Sequence alignment of histone H2A of different species. The highly conserved threonine 120 (Thr120) of H2A sequence in *C. japonica* is changed to either alanine (A) or serine (S). The translated sequence of three H2A transcripts identified from the *C. japonica* root transcriptomes were used in the alignment.

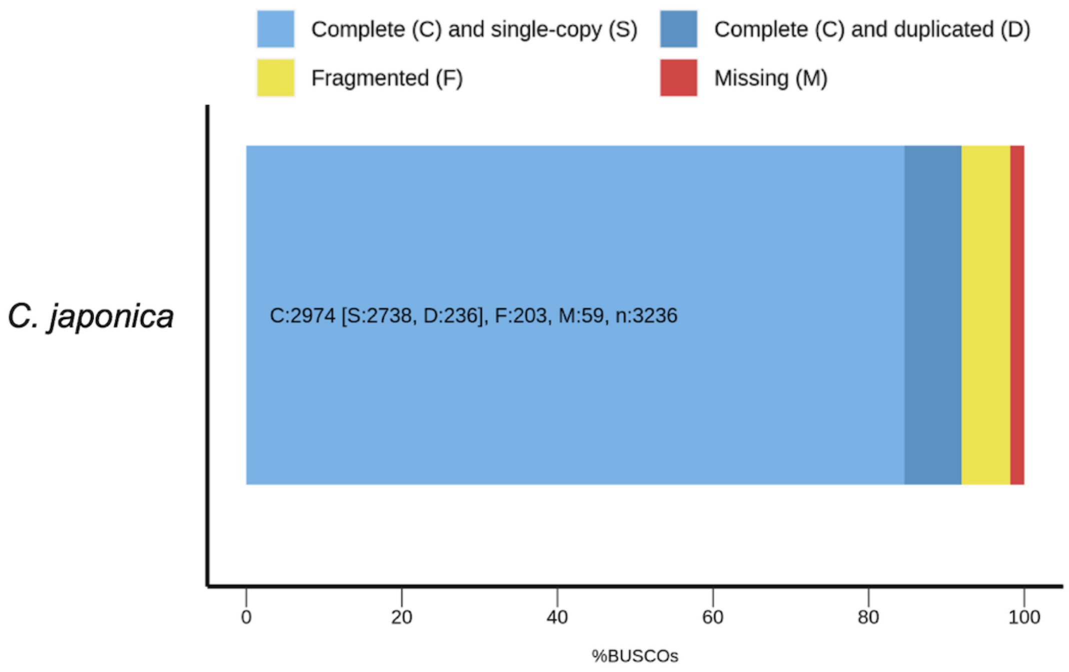

Supplementary Fig. 7.

BUSCO assessment of the *C. japonica* assembled genome. The completeness of the assembled genome was assessed using BUSCO analysis against the Liliopsida_odb10 dataset.

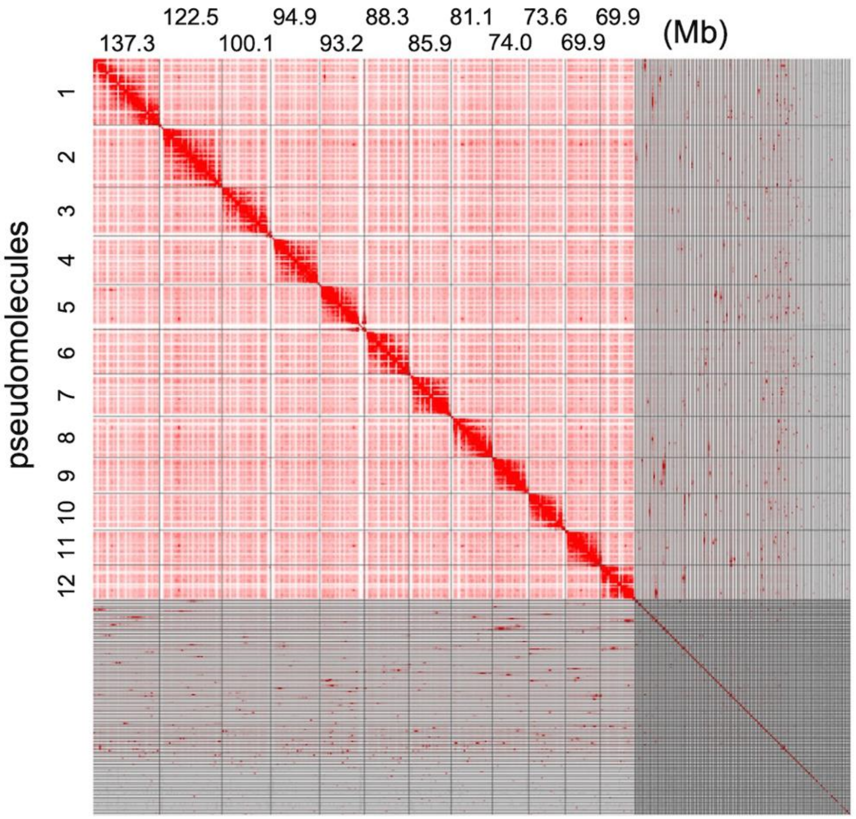

Supplementary Fig. 8.

Hi-C map for the assembled pseudomolecules and contigs of *C. japonica*. The size of the 12 pseudomolecules is indicated.

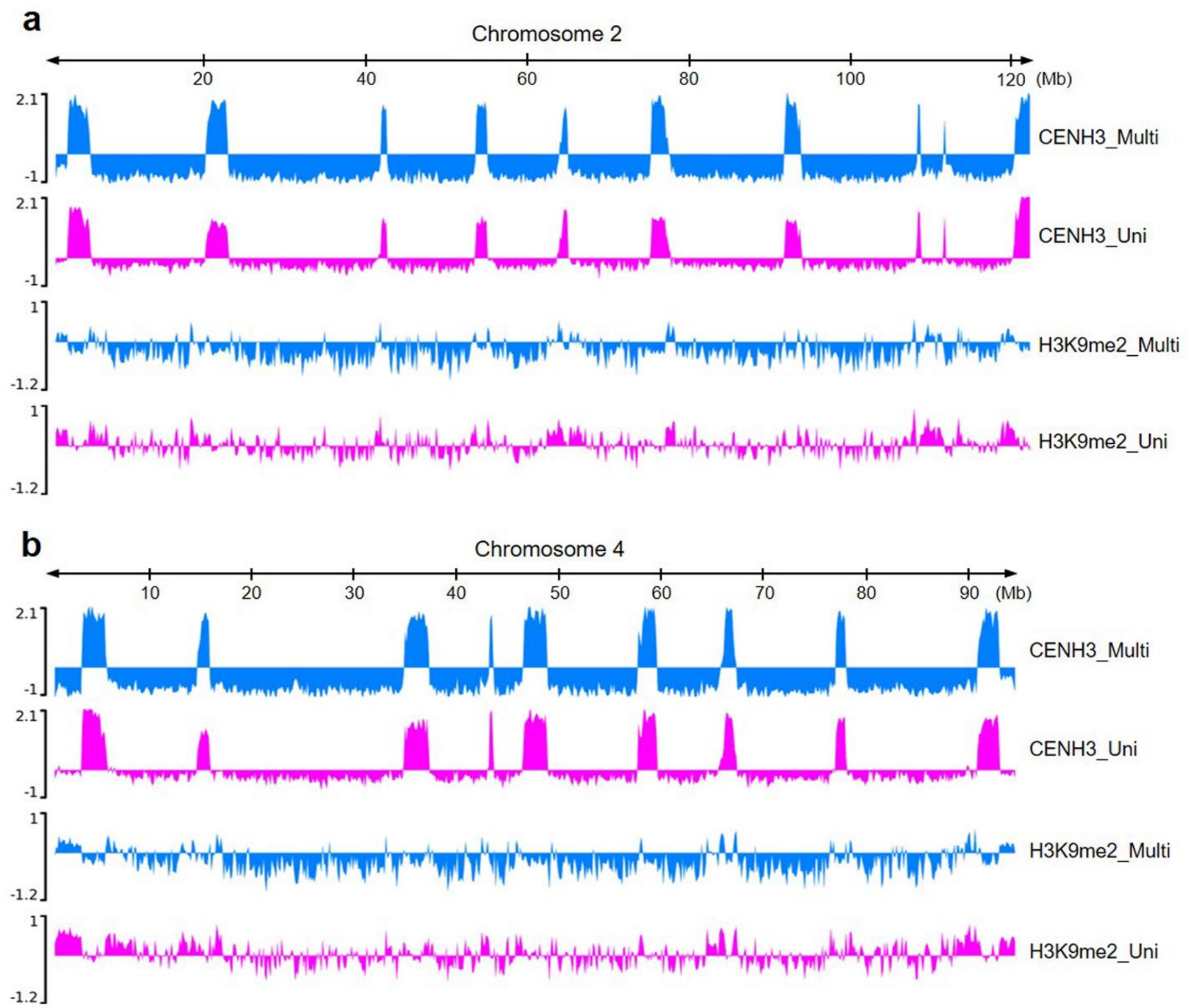

Supplementary Fig. 9.

Comparison of the CENH3- and H3K9me2-ChIPseq profiles generated using the multi- and uni-mapping modes. The uni-mapping patterns (Uni, tracks in magenta) were generated by filtering out all the multi-mapped reads in the Bowtie2 outputs using multi-mapping mode (Multi, tracks in blue). The $\log_2$ ChIP/Input ratio was calculated for the 1 kb window size. The CENH3- and H3K9me2-ChIPseq profiles analyzed using the multi-mapping mode in the (a) Chromosome 2 and (b) Chromosome 4 of *C. japonica* are corresponding to the tracks in Figure 4, 5, and Supplementary Fig. 12.

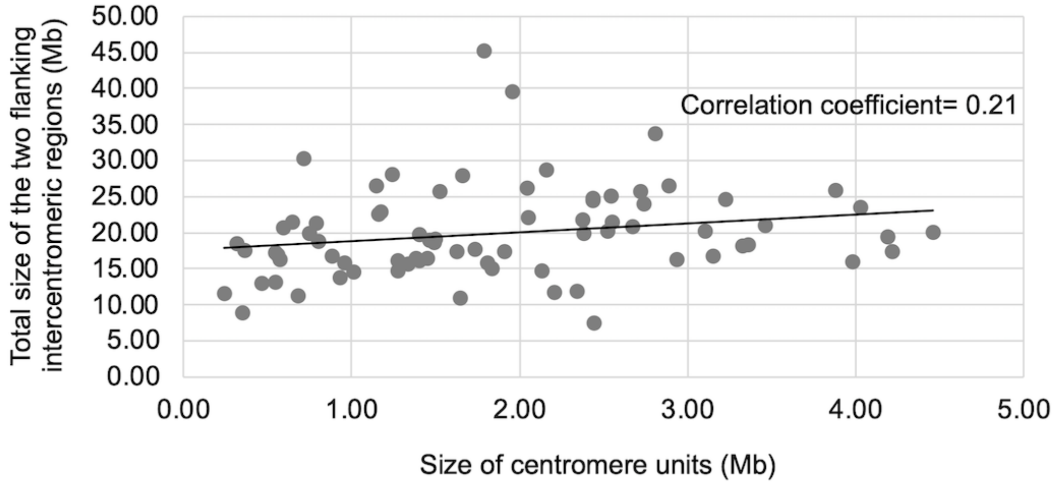

Supplementary Fig. 10.

Assessment of the correlation between the size of centromere units and two flanking intercentromeric regions. A low correlation was revealed with a correlation coefficient of 0.21. Source data are provided as a Source Data file.

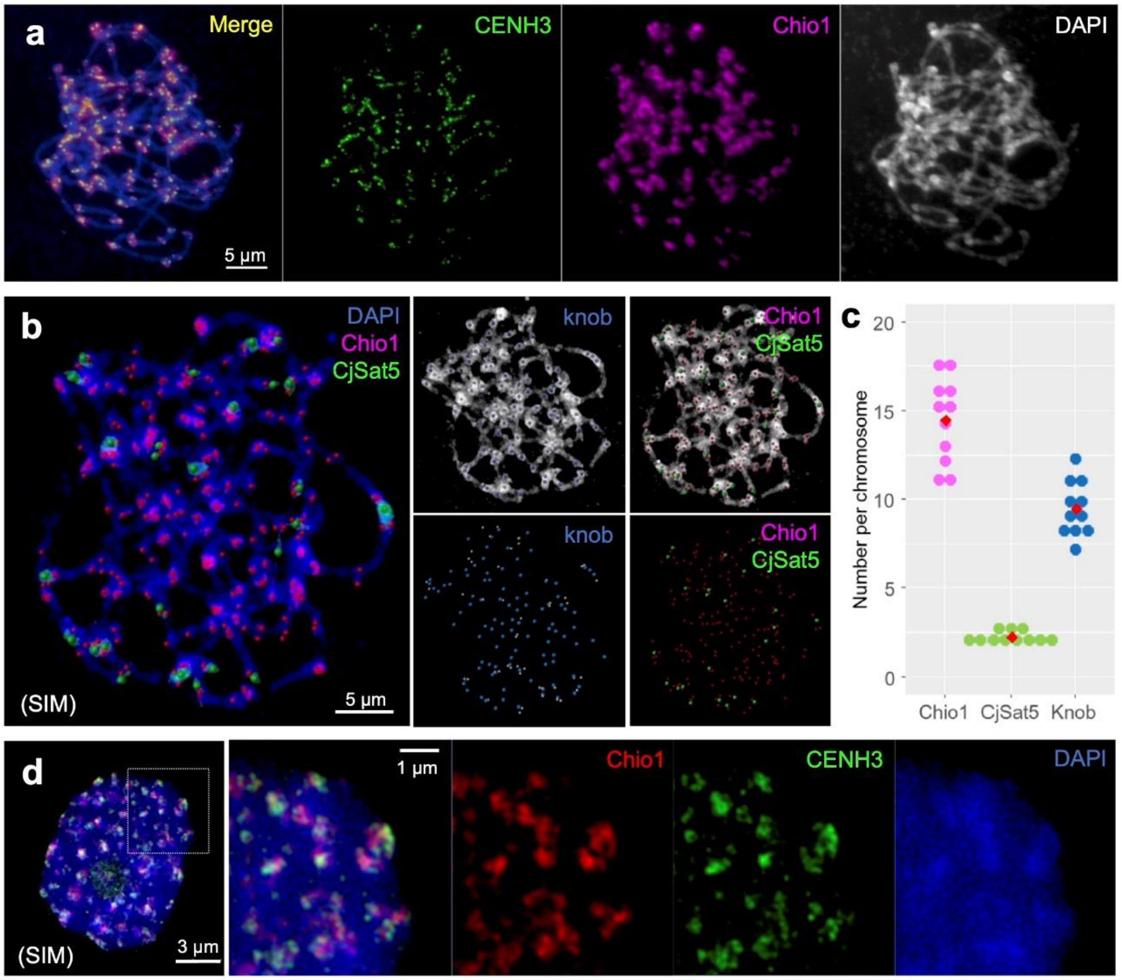

Supplementary Fig. 11.

The distribution of centromere units in *C. japonica*. (a) Immuno-FISH shows colocalization of the centromeric CENH3 (green) and Chio1 satellite repeat (purple) in pachytene chromosomes. (b) The knob structure (blue), signals of Chio1 (purple) and CjSat5 (green) satellite repeats were detected using the 'Spots' tool of Imaris 9.7, and (c) the average number of knobs, Chio1 and CjSat5 signals per chromosome were counted in 10 pachytene spreads (n=10). The red dots represent the average number. (d) The colocalization of CENH3 (green) and Chio1 (purple) in an interphase nucleus. The percentage of overlapped signals was ~65%, from 56.5% to 74.4% (n=11) measured by the Coloc tool of Imaris. Pachytene chromosomes and interphase nucleus were counterstained with DAPI. (a, b, d) At least two independent experiments were carried out to confirm the reproducibility of the labeling patterns. Source data are provided as a Source Data file.

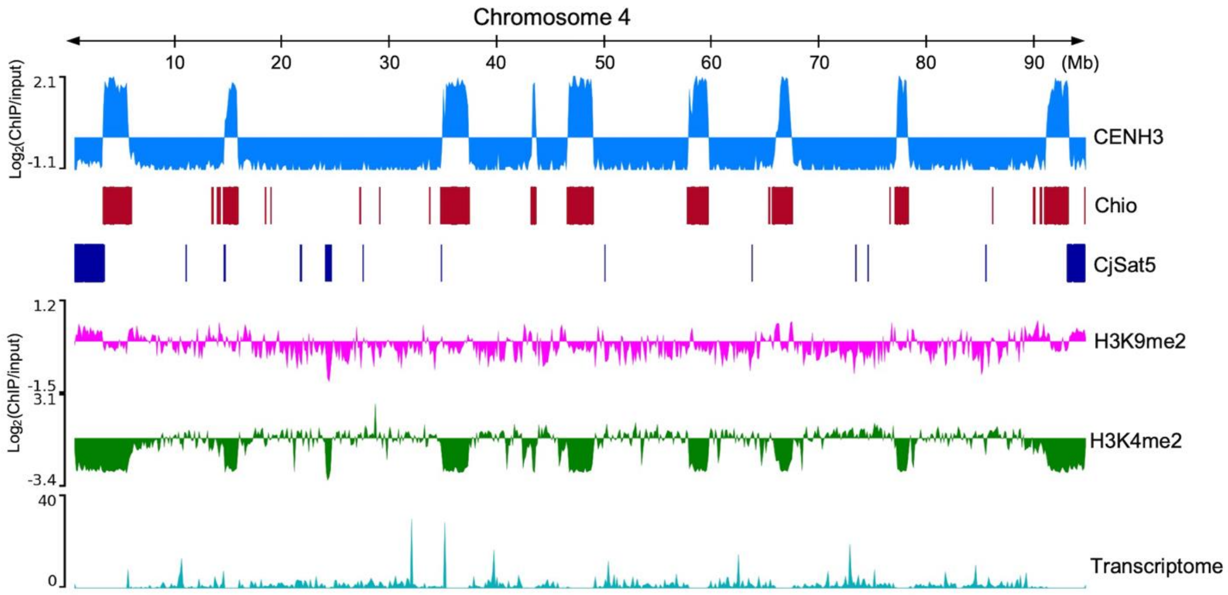

Supplementary Fig. 12.

The enrichment of CENH3-, H3K9me2-, and H3K4me2-ChIPseq and distribution of the centromeric Chio satellite arrays, subtelomeric CjSat5 satellite repeat, and root RNAseqs. The ChIPseq signal tracks are represented as the average of $\log_2$ ratio of ChIP/input in genome-wide 1 kb windows. Root transcriptome signal is shown as normalized read per kilobase per million (RPKM) in 1 kb windows.

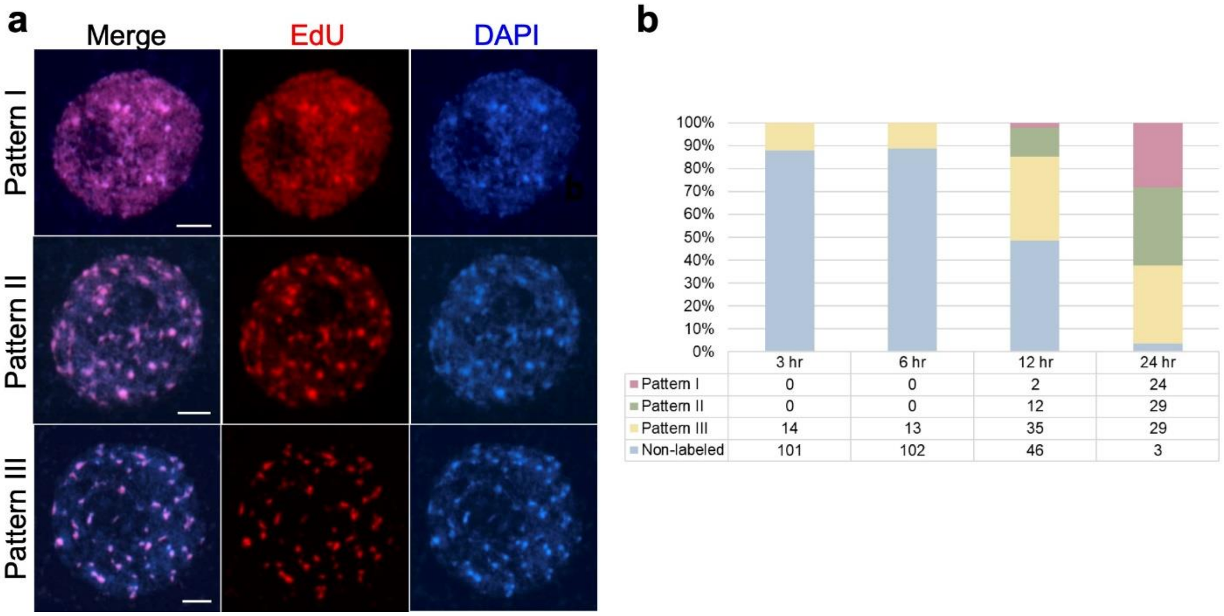

Supplementary Fig. 13.

EdU labeling-based DNA replication analysis of *C. japonica*. (a) The interphase replicating pattern I to III correspond to the early, mid, and late S phase, respectively. Scale bar, 5 μm. (b) The number of each pattern in the fixed materials with pulse recovery times of 3, 6, 12, and 24 hours counted in metaphase spreads are listed. The bar plot shows the percentage of each pattern in different samples and number of counted metaphase chromosome spreads is indicated in the table. (a) At least two independent experiments were carried out to confirm the reproducibility of the labeling patterns. Source data are provided as a Source Data file.

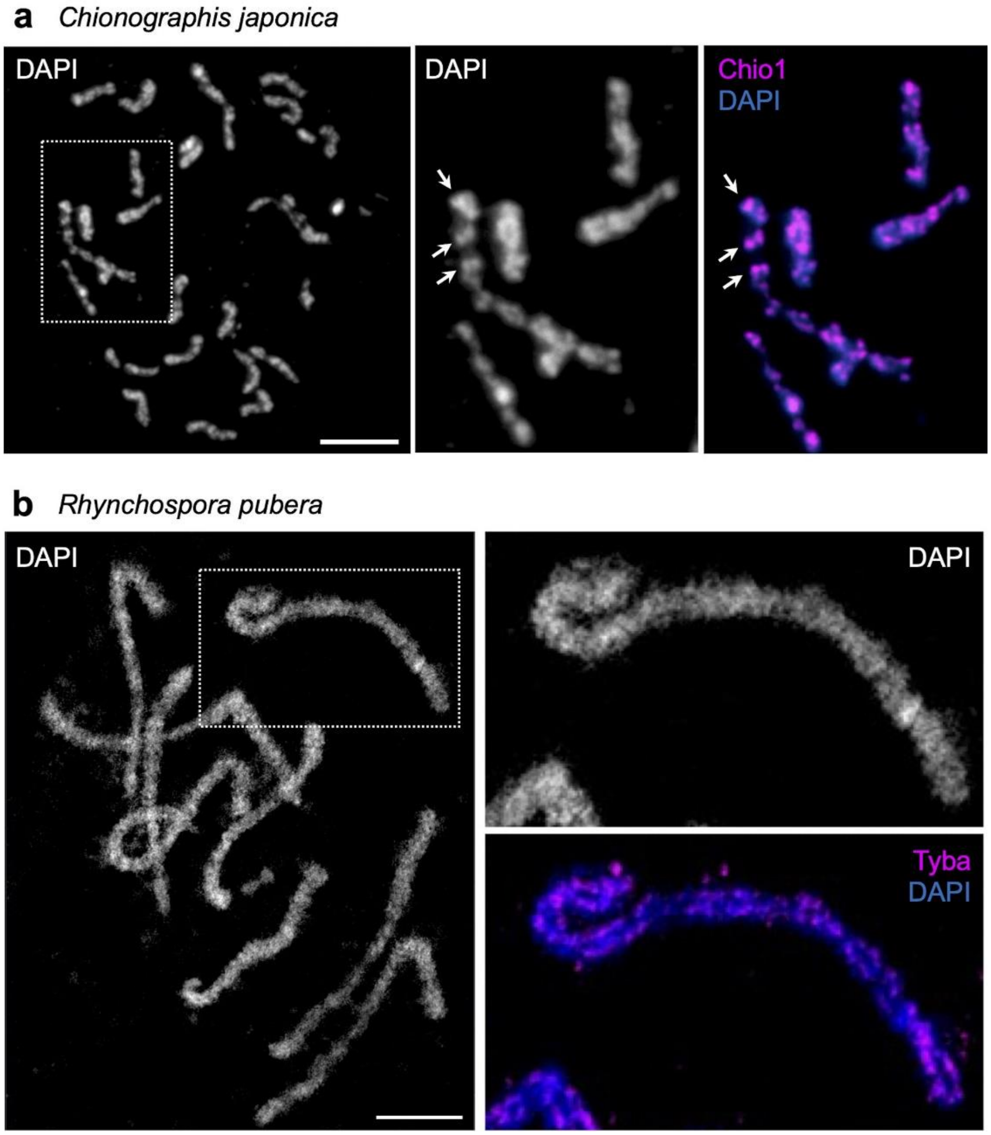

Supplementary Fig. 14.

Chromosome morphology of *C. japonica* and *R. pubera* during mitotic condensation. (a) The prometaphase chromosomes of *C. japonica* are non-uniformly condensed. Centromeric Chio1 repeats (purple) cluster and colocalize with heterochromatic regions. Enlargements (squares) are shown in the right panels. (b) In contrast, the prometaphase chromosomes of *R. pubera* show a uniform structure and line-like holocentromere-specific Tyba signals. Chromosomes were counterstained with DAPI. Scale bar, 5 µm. (a, b) At least two independent experiments were carried out to confirm the reproducibility of the labeling patterns.

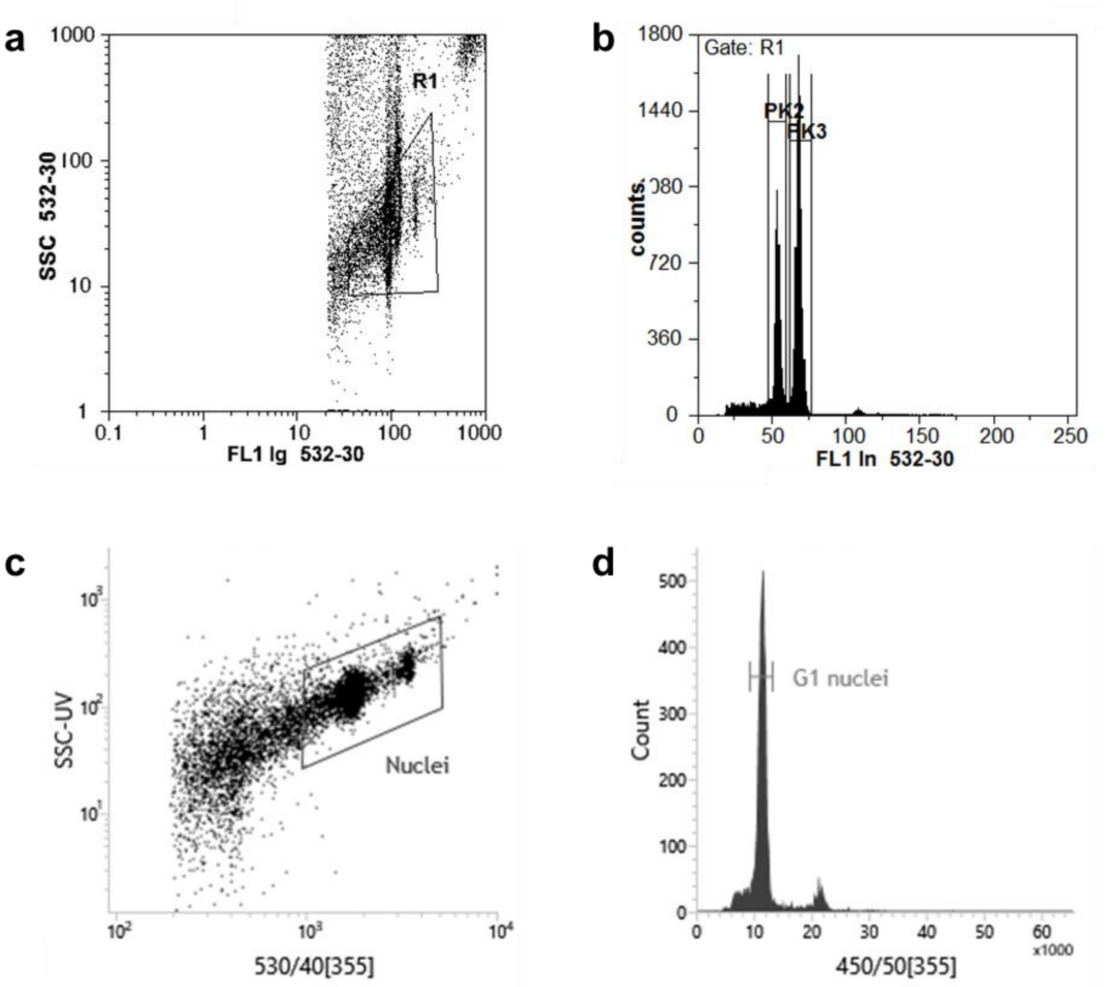

Supplementary Fig. 15

Applied gating strategy for genome size measurement using leaf nuclei (a, b) and sorting of G1 root meristem nuclei (c, d). The nuclei populations were separated from cellular debris by plotting the log-scale relative DNA content (a: propidium iodide (PI), c: DAPI) against log-scale SSC signals using either a green (a, b) or UV laser (c, d). (b) For genome size measurement, the mean fluorescence intensities of the G1 peaks of the sample (PK3) and reference (PK2) were determined using the ‘Find Peaks‘ function of the FloMax software in the corresponding histogram displaying the relative PI fluorescence intensity in lin-scale. (d) For sorting of G1 nuclei the corresponding peak was manually selected in a histogram displaying the lin-scale relative DAPI fluorescence intensity.

Supplementary Table 1

Number of CENH3 signal clusters in interphase nuclei of *C. japonica* (2*n* = 24) counted in 2D and 3D stacked images

Root G1 nuclei*	No. of CENH3 signal clusters per nucleus	No. of CENH3 signal clusters per chromosomes
2D image-1	64	2.67
2D image-2	49	2.04
2D image-3	74	3.08
2D image-4	82	3.42
2D image-5	79	3.29
2D image-6	72	3.00
2D image-7	57	2.38
2D image-8	59	2.46
2D image-9	59	2.46
2D image-10	101	4.21
2D image-11	70	2.92
2D image-12	50	2.08
2D image-13	107	4.46
2D image-14	76	3.17
2D image-15	76	3.17
2D image-16	94	3.92
2D image-17	68	2.83
2D image-18	62	2.58
2D image-19	80	3.33
2D image-20	58	2.42
2D image-21	71	2.96
2D image-22	58	2.42
2D image-23	79	3.29
2D image-24	67	2.79
2D image-25	70	2.92
2D image-26	49	2.04
2D image-27	48	2.00
2D image-28	55	2.29
2D image-29	63	2.63
2D image-30	48	2.00
**Average**	**68.17**	**2.85**
3D image-1	156	6.50
3D image-2	55	2.29
3D image-3	47	1.96
3D image-4	115	4.79
3D image-5	66	2.75
3D image-6	51	2.13
3D image-7	43	1.79
3D image-8	82	3.42
3D image-9	62	2.58
3D image-10	48	2.00
3D image-11	43	1.79
3D image-12	41	1.71
**Average**	**67.42**	**2.81**

* The G1 nuclei were isolated from roots of *C. japonica,* followed by sorting using flow cytometry.

Supplementary Table 2

Summary of the *de novo* genome assembly of *C. japonica*

	Chionographis japonica	
Genome size (Mb/1C)*	1,368	
GC content (%)	41.26	
Sequence coverage	58.5×	
Scaffolding strategy	Hi-C	
	Assembly	Scaffolding
Number of contigs/ scaffolds	3,786	3,263
Assembly size (bp)	1,526,137,861	1,526,120,517
Longest contig (bp)	11,690,282	137,288,582
N50 (bp)	2,877,649	81,106,678
N75 (bp)	1,067,322	2,104,563
L50	150	8
L75	373	32

*The genome size was determined by flow cytometry

Supplementary Table 3

Centromere characterization of the 12 chromosome scaffolds of *C. japonica.*

Chromosome scaffold	Total length (Mb)	No. of centromere units	Average size of centromere units (Mb)	Average interval between centromere units (Mb)
Chr 1	137.29	11	2.09	11.20
Chr 2	122.50	10	1.65	11.59
Chr 3	100.11	8	2.29	11.35
Chr 4	94.91	9	1.76	9.34
Chr 5	93.21	11	1.46	7.58
Chr 6	88.32	7	2.40	11.64
Chr 7	85.88	8	2.30	9.25
Chr 8	81.11	7	2.38	10.74
Chr 9	74.04	8	1.40	8.98
Chr 10	73.57	7	1.78	8.59
Chr 11	69.90	7	1.27	10.17
Chr 12	69.89	7	1.93	9.18
Average	90.89	8.3	1.89	9.97
Sum	1090.73	100	-	-

Supplementary Table 4

Characterization of high-copy satellite repeats used for FISH.

Satellite repeat	Monomer (bp)	Genome proportion (%)	Sequence of oligo probe/ PCR primer (5'-3')
Chio1	23	16.11	TCATTCGTACGATCCATTCTAAT
Chio2	28		TCATTCGTACGAATGTAGCCATTCTAAT
CjSat3	152	0.59	F: ACACCCTCTAAGAGCCTCGC<br>R: AGCCAAAACCGCTCCATATT
CjSat4	181	0.88	F: CGGACTTGCGAGCGAGTT<br>R: ATGCCGATCCGATGACGAT
CjSat5	270	2.20	F: CTCGACATGTTCGTGCTGAT<br>R: CCAGTCACAAGAAAACGGAGA
Tyba	172	-	F: CTAAGTCATTTCATCACAATAATCTAC<br>R: AATCCAGAAACGATTGAAATGCTC

Supplementary Table 5

Protocol for combined conventional and microwave-assisted fixation, dehydration and embedding in Spurr resin of root tips and leaf cuttings suitable for TEM.

Combined conventional & microwave assisted root tissue preparation in a PELCO Bio Wave®34700-230 (Ted Pella, Inc., Redding CA, USA)				
**Process**	**Reagent**	**Power [W]**	**Time [sec]**	**Vacuum [mm Hg]**
**1. Primary Fixation**	2.0% (v/v) glutaraldehyde and 2.0% (v/v) paraformaldehyde in 0.05 M cacodylate buffer (pH 7.3)	150<br>0<br>150<br>0<br>150	60<br>60<br>60<br>60<br>60	0<br>0<br>0<br>0<br>0
**2. Wash**	1x 0.05 M cacodylate buffer (pH 7.3) and 2x aqua dest.	150	60	0
**3. Secondary fixation**	1% (v/v) osmiumtetroxide in aqua dest.	0<br>80<br>0<br>80	60<br>120<br>60<br>120	10<br>10<br>10<br>10
	samples were kept for additional 15 min on a shaker			
**4. Wash**	3x aqua dest.	150	60	0
**5. Dehydration**	acetone: 30%, 40%, 50%, 60%, 70%, 80%, 90%, 2x 100% and 1 x propylenoxide.	150	60	0
	after each step samples were kept for additional 2 min on a shaker			
**6. Resin Infiltration**	25% Spurr resin in propylenoxide<br>50% Spurr resin in propylenoxide<br>75% Spurr resin in propylenoxide<br>100% Spurr resin	overnight on shaker at RT<br>4 hrs on shaker at RT<br>4 hrs on shaker at RT<br>overnight on shaker at RT		
**7. Polymerisation**	24 hrs at 70°C in prepolymerized flat embedding moulds in a heating cabinet			
