# Supplementary material for: Holocentromeres can consist of merely a few megabase-sized satellite arrays
Source: Nat Commun. 2023 Jun 13;14:3502. doi: 10.1038/s41467-023-38922-7 (PMC10264360; doi:10.1038/s41467-023-38922-7)
Supplement: Supplementary file 3 — Description of Additional Supplementary Files [file 41467_2023_38922_MOESM3_ESM.pdf]

Description of Additional Supplementary Files

File Name: Supplementary Movie 1
Description: Interaction of CENH3 (purple) and spindle microtubules (green) attachment sites along entire metaphase chromosomes of C. japonica. Rendering of 3D-SIM image stacks was performed using Imaris 9.7.

File Name: Supplementary Movie 2
Description: A single mitotic metaphase chromosome of C. japonica labeled with telomere (in green) and Chio 1 (in purple) probes. The telomere signals are at the chromosome ends, and the Chio1 clusters closely associated to each other, form a line-like holocentromere from telomere to telomere. At the end of the movie, the surface rendering shows a smooth surface of the metaphase chromosome. Unlike the holocentric plants L. elegans and R. pubera (Wanner et al. 2015), a chromosome groove was not detected in C. japonica. Rendering of 3D-SIM image stacks was performed using Imaris 9.7

File Name: Supplementary Movie 3
Description: Model of centromeric dynamics during the process of chromosome condensation in C. japonica. Centromere units cluster at interphase and form into a line-like holocentromere at prometaphase through chromosome condensation.
