# Supplementary material for: Holocentromeres can consist of merely a few megabase-sized satellite arrays
Source: Nat Commun. 2023 Jun 13;14:3502. doi: 10.1038/s41467-023-38922-7 (PMC10264360; doi:10.1038/s41467-023-38922-7)
Supplement: Supplementary file 7 — Reporting Summary [file 41467_2023_38922_MOESM7_ESM.pdf]

nature portfolio

Corresponding author(s): Yi-Tzu Kuo and Andreas Houben

Last updated by author(s): 2023.04.24

Reporting Summary

Nature Portfolio wishes to improve the reproducibility of the work that we publish. This form provides structure for consistency and transparency in reporting. For further information on Nature Portfolio policies, see our Editorial Policies and the Editorial Policy Checklist.

Statistics

For all statistical analyses, confirm that the following items are present in the figure legend, table legend, main text, or Methods section.

n/a	Confirmed	
☒	☐	The exact sample size (*n*) for each experimental group/condition, given as a discrete number and unit of measurement
☒	☐	A statement on whether measurements were taken from distinct samples or whether the same sample was measured repeatedly
☐	☒	The statistical test(s) used AND whether they are one- or two-sided<br>*Only common tests should be described solely by name; describe more complex techniques in the Methods section.*
☐	☒	A description of all covariates tested
☐	☒	A description of any assumptions or corrections, such as tests of normality and adjustment for multiple comparisons
☐	☒	A full description of the statistical parameters including central tendency (e.g. means) or other basic estimates (e.g. regression coefficient) AND variation (e.g. standard deviation) or associated estimates of uncertainty (e.g. confidence intervals)
☐	☒	For null hypothesis testing, the test statistic (e.g. *F*, *t*, *r*) with confidence intervals, effect sizes, degrees of freedom and *P* value noted<br>*Give P values as exact values whenever suitable.*
☐	☒	For Bayesian analysis, information on the choice of priors and Markov chain Monte Carlo settings
☐	☒	For hierarchical and complex designs, identification of the appropriate level for tests and full reporting of outcomes
☐	☒	Estimates of effect sizes (e.g. Cohen's *d*, Pearson's *r*), indicating how they were calculated

Our web collection on statistics for biologists contains articles on many of the points above.

Software and code

Policy information about availability of computer code

Data collection	Genome size measurement was performed using a Sysmex CyFlow Space flow cytometer (Sysmex-Partec, Germany). Sorting of G1 nuclei was performed by a BD Influx cell sorter (BD Biosciences). Fluorescence-activated cell sorting was performed with a BD Influx™ (BD Biosciences) cell sorters. The Image of Western blot was captured using Odyssey (Li-Cor). The Epifluorescence microscope BX61 (Olympus, Germany), Elyra PS.1 microscope (Carl Zeiss), and Transmission Electron Microscope Tecnai Sphera G2 120 kV (FEI) were used to take microscopic images.

Data analysis	Genome size measurement: FloMax Operating and Analysis Software for Flow Cytometry Particle Analysing Systems, Version 2.82; Sorting of G1 nuclei: BD FACS Sortware Version 1.2.0.142. FastQC Galaxy Version 0.72. The images analyzed were performed using the Zeiss ZENBlack (Carl Zeiss), Imaris 9.6 and 9.7 (Bitplane), Adobe Photoshop 6.0 (Adobe), Image J softwares. Hifiasm assembler Version 0.15.3 (https://github.com/chhylp123/hifiasm) was used for genome assembly; hic-pipeline (https://github.com/esrice/hic-pipeline), SALSA2 (https://github.com/marbl/SALSA), MAPQ10, and BWA Version 0.7.17.4, were applied for Hi-C scaffolding. BUSCO Galaxy Version 5.4.5 (https://plants.usegalaxy.eu/) was used to assess the genome assembly. Genome repetitive analysis was carried out using RepeatExplorer2 Galaxy Version 2.3.8.1, DANTE (https://github.com/kavonrtep/dante) Galaxy Version 1.1.0, DANTE-LTR tools (https://github.com/kavonrtep/dante_ltr) and TAREAN Galaxy Version 2.3.8.1 implemented in Galaxy server (https://repeatexplorer-elixir.cerit-sc.cz/galaxy/), ChIPseq datasets were analyzed using ChIP-Seq Mapper Galaxy Version 0.1.1 (https://repeatexplorer-elixir.cerit-sc.cz/galaxy/), Bowtie2 Galaxy Version 2.3.4.3 (https://galaxy.ipk-gatersleben.de/), Sambamba 1.0.0 (https://lomereiter.github.io/sambamba/), MACS3 (https://github.com/macs3-project/MACS), epic2 (https://github.com/biocore-ntnu/epic2), bedtools Version 2.30.0, and Deeptools Galaxy Version 3.3.0.0.0. Transcriptomic datasets were analyzed using Trinity 2.4.0 and NCBI BLASTtools Galaxy Version 2.10.1. DNA methylation was evaluated using Bismarck Version 0.22.1(https://github.com/FelixKrueger/Bismark). Genome browser tracks were produced using pyGenomeTracks Galaxy Version 3.8 (https://github.com/deeptools/pyGenomeTracks). Polymer simulation was carried out using OpenMM Python API Version 8.0.0 (http://docs.openmm.org/latest/api-python/) and PyMOL Version 2.1.1 (https://pymol.org/2/).

For manuscripts utilizing custom algorithms or software that are central to the research but not yet described in published literature, software must be made available to editors and reviewers. We strongly encourage code deposition in a community repository (e.g. GitHub). See the Nature Portfolio guidelines for submitting code & software for further information.

Data

Policy information about availability of data

All manuscripts must include a data availability statement. This statement should provide the following information, where applicable:

- Accession codes, unique identifiers, or web links for publicly available datasets
- A description of any restrictions on data availability
- For clinical datasets or third party data, please ensure that the statement adheres to our policy

The data generated in this study have been deposited in the European Nucleotide Archive (ENA) at EMBL-EBI under the umbrella project no. PRJEB58432 [https://www.ebi.ac.uk/ena/browser/view/PRJEB58432]. The processed ChIP-seq datasets are at the NCBI GEO database under the accession code GSE228407 [https://www.ncbi.nlm.nih.gov/geo/query/acc.cgi?acc=GSE228407]. Source data are provided with this paper. The REXdb database Viridiplantae v3.0 used in this study is publicly available [http://repeatexplorer.org/?page_id=918].

Human research participants

Policy information about studies involving human research participants and Sex and Gender in Research.

Reporting on sex and gender	N/A
Population characteristics	N/A
Recruitment	N/A
Ethics oversight	N/A

Note that full information on the approval of the study protocol must also be provided in the manuscript.

Field-specific reporting

Please select the one below that is the best fit for your research. If you are not sure, read the appropriate sections before making your selection.

☒ Life sciences ☐ Behavioural & social sciences ☐ Ecological, evolutionary & environmental sciences

For a reference copy of the document with all sections, see nature.com/documents/nr-reporting-summary-flat.pdf

Life sciences study design

All studies must disclose on these points even when the disclosure is negative.

Sample size	We decided the sample size based on available literatures in the field, our own experience in previous studies, and requirement for corresponding protocols. The sample sizes used for all experiments provided sufficient resolving power.
Data exclusions	No data was excluded from the analysis.
Replication	The number of replication are indicated in the section Methods and Figure legends. For fluorescence in situ hybridization and immunostaining, at least two independent experiments were carried out to confirm the reproducibility of the labeling patterns. For histone ChIPseq, two replicates were performed and the the correlation between independent experiment was checked. All replications were

successful.

Randomization

A randomization is not relevant for this study because no genotype or treatment were compared with each other. Hower, the tissues for cytogenetic and ChIPseq experiments were randomly collected from different plant individuals grown under the same condition in a greenhouse. Only for genome assembly, we used the HMW DNA from a single Chionographis japonica plant for long-read sequencing, to avoid problems due to the highly heterozygous genome of C. japonica.

Blinding

All the experiments were performed without prior knowledge of the final outcome, and therefore blinding was not applied.

Reporting for specific materials, systems and methods

We require information from authors about some types of materials, experimental systems and methods used in many studies. Here, indicate whether each material, system or method listed is relevant to your study. If you are not sure if a list item applies to your research, read the appropriate section before selecting a response.

Materials & experimental systems

n/a	Involved in the study
☐	☒ Antibodies
☒	☐ Eukaryotic cell lines
☒	☐ Palaeontology and archaeology
☒	☐ Animals and other organisms
☒	☐ Clinical data
☒	☐ Dual use research of concern

Methods

n/a	Involved in the study
☐	☒ ChIP-seq
☐	☒ Flow cytometry
☒	☐ MRI-based neuroimaging

Antibodies

Antibodies used

Customized Chionographis japonica-specific antibodies:
rabbit anti-CjCENH3
rabbit anti-CjMIS12
rabbit anti-CjNDC80.

Commercially available antibodies:
mouse anti-alpha-tubulin (Sigma-Aldrich, USA, cat. no. T9026-2, dilution 1:300)
rabbit anti-histone H3K4me2 (abcam, UK, cat. no. ab7766, dilution 1:300)
mouse anti-histone H3K9me2 (abcam, UK, cat. no. ab1220, dilution 1:200)
mouse anti-histone H3S10ph (abcam, UK, cat. no. ab14955, dilution 1:1000)
rat anti-histone H3S28ph (Sigma-Aldrich, USA, cat. no. H9908, dilution 1:1000)
mouse anti-H3T3ph (Sigma-Aldrich, USA, cat. no. 07-424, dilution 1:1000)
rabbit anti-H2AT120ph (Active Motif, USA, cat. no. 61196, dilution 1:500)
anti-rabbit rhodamine (Jack ImmunoResearch, USA, cat. no. 111-295-144, dilution 1:400)
anti-rabbit Alexa488 (Jack ImmunoResearch, USA, cat. no. 711-545-152, dilution 1:400)
anti-mouse Alexa488 (Jack ImmunoResearch, USA, cat. no. 715-546-151, dilution 1:400)
anti-rat Alexa488 (Jack ImmunoResearch, USA, cat. no. 112-545-167, dilution 1:400)
anti-rabbit IRDye 800CW (LI-COR, USA, cat. no. NC9401842, dilution 1:5000)

Validation

Validation by commercial providers:
mouse anti-alpha-tubulin (Sigma-Aldrich, USA, cat. no. T9026-2), https://www.sigmaaldrich.com/DE/de/product/sigma/t9026?gclid=EAIaIQobChMIgZ3Z0_my_gIVEepRCh2ZzAYvEAAYASAAEgKmOfD_BwE&gclsrc=aw.ds
rabbit anti-histone H3K4me2 (abcam, UK, cat. no. ab7766), https://www.abcam.com/products/primary-antibodies/histone-h3-di-methyl-k4-antibody-chip-grade-ab7766.html
mouse anti-histone H3K9me2 (abcam, UK, cat. no. ab1220), https://www.abcam.com/products/primary-antibodies/histone-h3-di-methyl-k9-antibody-mabcam-1220-chip-grade-ab1220.html
mouse anti-histone H3S10ph (abcam, UK, cat. no. ab14955), https://www.abcam.com/products/primary-antibodies/histone-h3-phospho-s10-antibody-mabcam-14955-ab14955.html
rat anti-histone H3S28ph (Sigma-Aldrich, USA, cat. no. H9908), https://www.sigmaaldrich.com/DE/de/product/sigma/h9908
mouse anti-H3T3ph (Sigma-Aldrich, USA, cat. no. 07-424), https://www.merckmillipore.com/DE/de/product/Anti-phospho-Histone-H3-Thr3-Antibody,MM_NF-07-424?ReferrerURL=https%3A%2F%2Fwww.google.com%2F
rabbit anti-H2AT120ph (Active Motif, USA, cat. no. 61196)
https://www.activemotif.com/catalog/details/61195/histone-h2a-phospho-thr120-antibody-pab-1
anti-rabbit rhodamine (Jack ImmunoResearch, USA, cat. no. 111-295-144), https://www.jacksonimmuno.com/catalog/products/111-295-144
anti-rabbit Alexa488 (Jack ImmunoResearch, USA, cat. no. 711-545-152), https://www.jacksonimmuno.com/catalog/products/711-545-152
anti-mouse Alexa488 (Jack ImmunoResearch, USA, cat. no. 715-546-151), https://www.jacksonimmuno.com/catalog/products/715-546-151
anti-rat Alexa488 (Jack ImmunoResearch, USA, cat. no. 112-545-167), https://www.jacksonimmuno.com/catalog/products/112-545-167
anti-rabbit IRDye 800CW (LI-COR, USA, cat. no. NC9401842), https://www.licor.com/bio/reagents/irdye-800cw-goat-anti-rabbit-igg-

secondary-antibody?utm_source=google&utm_medium=adwords&utm_content=reagent-webpage&utm_campaign=reagents&gclid=EAIaIQobChMI86qv9P2y_gIV0bHVCh0ocQe2EAAYASAAEgL7gPD_BwE

The customized C. japonica-specific peptide rabbit anti-CENH3 antibody generated by the company LifeTein was validated by Western blot, as described in the section Methods and the result shown in Supplementary Fig. 2.
The customized peptide rabbit C. japonica anti-MIS12 and anti-NDC80 antibodies generated by the company LifeTein were validated by peptide ELISA tests. The ELISA information is available upon request. The observed indirect immunosignals of anti-MIS12 and anti-NDC80 were the same as reported in the published literature for other species.

ChIP-seq

Data deposition

☒ Confirm that both raw and final processed data have been deposited in a public database such as GEO.

☒ Confirm that you have deposited or provided access to graph files (e.g. BED files) for the called peaks.

Data access links
May remain private before publication.

The raw ChIPseq data were deposited in the European Nucleotide Archive (ENA) at EMBL-EBI, accession number PRJEB58126 (Chionographis_japonica_ChIP-Seq) under the umbrella project no. PRJEB58432 [https://www.ebi.ac.uk/ena/browser/view/PRJEB58432]. The processed data were deposited in the NCBI GEO database under the accession number GSE228407 [https://www.ncbi.nlm.nih.gov/geo/query/acc.cgi?acc=GSE228407].

Files in database submission

CENH3-ChIPseq datasets:
CENH3_1kb 1st repllicate_Multi.bigwig (The first biological replicate mapping by using multiple-mapping mode)
CENH3_1kb 2nd repllicate_Multi.bigwig (The second biological replicate mapping by using multiple-mapping mode)
CENH3_1kb 1st repllicate_Uni.bigwig (The first biological replicate mapping by using unique-mapping mode)

H3K4me2-ChIPseq datasets:
H3K4me2_1kb 1st replicate_Multi.bigwig (The first biological replicate mapping by using multiple-mapping mode)
H3K4me2_1 kb 2nd replicate_Multi.bigwig (The second biological replicate mapping by using multiple-mapping mode)

H3K9me2-ChIPseq datasets:
H3K9me2_1kb 1st repllicate_Multi.bigwig (The first biological replicate mapping by using multiple-mapping mode)
H3K9me2_1kb_1st replicate_Uni.bigwig (The first biological replicate mapping by using unique-mapping mode)
H3K9me2_1kb_2nd replicate_Multi.bigwig (The second biological replicate mapping by using multiple-mapping mode)
H3K9me2_1kb_2nd replicate_Uni.bigwig (The second biological replicate mapping by using unique-mapping mode)

Genome browser session
(e.g. UCSC)

No longer applicable

Methodology

Replicates

All CENH3-, H3K9me2- and H3K4me2-ChIPseq experiments were performed for two biological replicates.

Sequencing depth

For all biological replicates of CENH3-, H3K9me2- and H3K4me2-ChIPseq experiments, at least 6 Gb of paired-end 150 bp illumina raw reads were generated.

Antibodies

Chionographis japonica-specific rabbit anti-CjCENH3 antibody, the commercially available rabbit anti-histone H3K4me2 (abcam, UK, cat. no. ab7766), and mouse anti-histone H3K9me2 (abcam, UK, cat. no. ab1220).

Peak calling parameters

The read mapping was performed by Bowtie2 with default setting, and the multimapped reads were filtered out from teh Bowtie2 outputs using Sambamba with options "-F [XS] == null and not unmapped and not duplicate". The CENH3 domains were identified by comparing the ChIP and input data using MACS3 with parameters "-B –broad –g 1380000000 –trackline" and also alternatively using epic2 with parameters included "--bin-size 2000". The Deeptools bamCompare was used to generate normalized ChIPseq signal tracks of the average of log2-ratio of read counts in ChIP over input.

Data quality

The two biological replicates for each anti-histone ChIPseq with the Spearman correlation coefficient >0.89 were retained. Visualization of the each ChIPseq profile on genome browser showed similar distribution patterns with only minor differences. We further confirmed that similar genome profiles of the ChIPseq experiments can be obtained using either multi-mapping or unique-mapping modes.

Software

ChIPseq datasets were analyzed using ChIP-Seq Mapper Galaxy Version 0.1.1 (https://repeatexplorer-elixir.cerit-sc.cz/galaxy/), Bowtie2 Galaxy Version 2.3.4.3 (https://galaxy.ipk-gatersleben.de/), Sambamba (https://lomereiter.github.io/sambamba/), MACS3 (https://github.com/macs3-project/MACS), epic2 (https://github.com/biocore-ntnu/epic2), and Deeptools Galaxy Version 3.3.0.0.0.

Flow Cytometry

Plots

Confirm that:

☒ The axis labels state the marker and fluorochrome used (e.g. CD4-FITC).

☒ The axis scales are clearly visible. Include numbers along axes only for bottom left plot of group (a 'group' is an analysis of identical markers).

☒ All plots are contour plots with outliers or pseudocolor plots.

☒ A numerical value for number of cells or percentage (with statistics) is provided.

Methodology

Sample preparation	Genome size measurement: Nuclei were isolated by manual chopping of young leaf tissue using a sharp razor blade in nuclei isolation buffer (CyStain PI Absolute P; Sysmex-Partec) and subsequent filtering through a 50 µm mesh (CellTrics, Sysmex-Partec).<br>Sorting of G1 nuclei: To isolate meristematic nuclei, the meristems of roots fixed in 4% paraformaldehyde were cut off and chopped with a sharp razor blade in LB01 nuclei isolation buffer (Dolezel et al., 1989) and subsequently filtered through a 50 µm mesh (CellTrics, Sysmex-Partec).
Instrument	Genome size measurement: Sysmex CyFlow Space flow cytometer (Sysmex-Partec, Germany); Sorting of G1 nuclei: BD Influx cell sorter (BD Biosciences)
Software	Genome size measurement: FloMax Operating and Analysis Software for Flow Cytometry Particle Analysing Systems, Version 2.82; Sorting of G1 nuclei: BD FACS Sortware ver. 1.2.0.142
Cell population abundance	Genome size measurement: Based on the applied threshold setting (590LP) for the green laser (532 nm) the abundance of nuclei within the suspensions was above 70 %.<br>Sorting of G1 nuclei: Based on the applied threshold setting (530/40) for the UV laser (355 nm) the abundance of nuclei within the suspensions was above 65 %, of those around 80 % were identified as G1 nuclei.
Gating strategy	Genome size measurement: Nuclei were separated from cellular debris by plotting the log-scale relative DNA content (propidium iodide, 590 LP) against log-scale SSC signals (532/30) using a green laser (532 nm) for excitation.<br>Sorting of G1 nuclei: Nuclei were separated from cellular debris by plotting the log-scale relative DNA content (DAPI, 530/40) against log-scale SSC signals (532/30) using a UV laser (355 nm) for excitation. The sorting gate was defined in a histogram displaying lin-scale relative DNA content (DAPI, 450/50).

☒ Tick this box to confirm that a figure exemplifying the gating strategy is provided in the Supplementary Information.
